# IL‐22 sustains epithelial integrity in progressive kidney remodeling and fibrosis

**DOI:** 10.14814/phy2.13817

**Published:** 2018-08-28

**Authors:** Marc Weidenbusch, Shangqing Song, Takamasa Iwakura, Chongxu Shi, Severin Rodler, Sebastian Kobold, Shrikant R. Mulay, Mohsen M. Honarpisheh, Hans‐Joachim Anders

**Affiliations:** ^1^ Medizinische Klinik und Poliklinik IV Klinikum der Universität München Ludwig Maximilians University of Munich Munich Germany; ^2^ Department of Urology Shanghai Ninth People's Hospital Shanghai Jiaotong University School of Medicine Shanghai China; ^3^ Center for Integrated Protein Science Munich (CIPSM) and Abteilung für Klinische Pharmakologie Medizinische Klinik und Poliklinik IV Klinikum der Universität München Ludwig Maximilians University of Munich Munich Germany

**Keywords:** IL‐22, kidney, tubular cell injury, interstitial fibrosis

## Abstract

IL‐22, a member of the IL‐10 cytokine family, accelerates tubule regeneration upon acute kidney injury, hence we speculated on a protective role also in chronic kidney disease. We quantified intrarenal IL‐22 expression after unilateral ureteral (UUO) in wild‐type mice and performed UUO in IL‐22 knock‐out animals. Obstruction phenotypic differences between *IL22* ^+/+^ and *IL22* ^−/−^ mice were assessed by histology, immunohistochemistry, immunofluorescence as well as western blotting and reverse‐transcriptase quantitative PCR *ex vivo*. Additionally, we performed *in vitro* experiments using both murine and human tubular cells to characterize IL‐22 effects in epithelial healing. We found increasing IL‐22 positivity in infiltrating immune cells over time upon UUO in wild‐type mice. UUO in *IL22* ^−/−^ mice caused more tubular cell injury as defined by TUNEL positive cells and loss of *tetragonolobus* lectin staining. Instead, tubular dilation, loss of CD31+ perivascular capillaries, and interstitial fibrosis were independent of the *Il22* genotype as assessed by standard histology, immunostaining, and mRNA expression profiling. *In vitro* experiments showed that recombinant human IL‐22 significantly enhanced human tubular epithelial cell proliferation and wound closure upon mechanical injury, and electric cell‐substrate impedance sensing studies revealed that recombinant IL‐22 sustained tubular epithelial barrier function upon injury. In contrast, IL‐22 had no such direct effects on human fibroblasts. Together, in progressive kidney remodeling upon UUO, infiltrating immune cells secrete IL‐22, which augments tubular epithelial integrity and epithelial barrier function, but does not affect vascular rarefaction or fibrogenesis. We conclude that IL‐22 could represent a molecular target to specifically modulate tubular atrophy.

## Introduction

Acute kidney injury (AKI) and its long‐term consequence, chronic kidney disease (CKD), are increasing global health concerns (Levin et al. 2017; Romagnani et al. 2017). Both AKI and CKD are severe disorders with a growing number of patients and associated with high morbidity as well as mortality. While there is a plethora of initial injurious triggers (ischemic, toxic, inflammatory, obstructive injury, etc.), most of these processes lead to inflammation and cell death, a phenomenon dubbed “necroinflammation”. Eventually, this leads to progressive nephron loss and renal scaring/fibrosis, as tubular cells are replaced by mesenchymal tissue. Consistent with the central role of immune cells in AKI, we and others have shown a reno‐protective role of IL‐22 (Kulkarni et al. 2014; Xu et al. 2014), balancing concurrent detrimental intrarenal inflammation. Nevertheless, it is unknown whether IL‐22 also plays a role in subacute or chronic kidney disease, where typically there is less inflammation.

Interleukin‐22 (IL‐22) is a member of the IL‐10 family of cytokines produced by several subsets of lymphocytes such as T helper (Th) 17 cells, NKT cells, *γδ*T cells, and innate lymphoid cells (ILCs) (Dudakov et al. 2015; Weidenbusch et al. 2015). It has been proven that IL‐22 is expressed constitutively in a broad array of tissues, including thymus, brain, gut, skin, spleen, pancreas, liver, lung and kidney (Weidenbusch et al. 2017). IL‐22 binds to a class II cytokine receptor (IL‐22R) composed of IL‐22RA1 and IL‐10RB2 subunits leading to the activation of signal transducer and transcription factor 3 (STAT3)‐dependent downstream signaling pathways (Li et al. 2004). IL‐22R is mainly expressed by epithelial cells in a variety of parenchymal organs, but absent on immune cells, hence establishing a means of “immune‐epithelial” signaling. In general, IL‐22 acts on epithelia cells to enhance epithelial barrier functions, is involved both in tissue homeostasis as well as wound healing/tissue repair.

Unilateral ureteral obstruction (UUO) reproduces obstructive nephropathy in humans including tubular epithelial cell loss, immune cell infiltration, and interstitial fibrosis (Ucero et al. 2014; Guiteras et al. 2017; Liu et al. 2017; Qiao et al. 2017; Xiao et al. 2017). Furthermore, UUO mimics the morphological features of CKD progression in terms of progressive nephron loss, kidney atrophy, and renal scaring.

We hypothesized that intrarenal leukocyte‐derived IL‐22 would augment tubule integrity also in progressive obstructive nephropathy. To test this concept, we employed a series of *in vitro* and *in vivo* studies including UUO surgeries in *Il22*‐deficient mice and experiments with recombinant IL‐22 and human or murine renal parenchymal cells.

## Material and methods

### Animal experiments

*Il22*‐deficient mice in the BALB/cJ genetic background were generated by Genentech as described (Zheng et al. 2007). BALB/cByJ wild‐type mice were obtained from Charles River (Sulzfeld, Germany) as controls. All mice were housed under SPF conditions in groups of 5 mice in filter top cages with a 12‐h dark‐light cycle and unlimited access to food and water. Cages, nestles, bedding, food, and water were autoclaved for sterilization before use. UUO was performed in 8–12 weeks old, sex‐ and age‐matched wild‐type and *Il22*‐deficient mice. After general anesthesia, the ureteric obstruction was performed by ligating the left distal ureter with 4‐0 Mersilene suture through a low midline abdominal incision and unobstructed contralateral kidneys were used as intraindividual controls as described(Higgins et al. 2003; Skuginna et al. 2011). Mice were sacrificed in deep anesthesia by cervical dislocation and both sides of the kidneys were harvested at 1 day, 5 days or 10 days (n = 5–7 in each group) after UUO surgery. Kidneys were then cut into three pieces, using one part each for histological, western blotting, and gene expression analyses. All experiments were conducted according to German animal protection laws and had been approved by the local government authorities.

### Histological evaluation

Kidneys were fixed in 4% neutral‐buffered formalin and embedded in paraffin. 2 *μ*m sections were used for Silver stains and immune staining as described (Mulay et al. 2012). Kidney injury and fibrosis were identified by silver staining (Bio‐Rad, California, USA). CD31 (Dianova GmbH, Hamburg, Germany) staining was used to demonstrate the presence of endothelial cells in kidney sections.

*L. tetragonolobus* lectin (Vector Labs, California, USA) stainings were used to quantify proximal renal tubular cell mass and terminal‐deoxynucleotidyl transferase‐mediated digoxigenin‐deoxyuridine nick‐end labeling (TUNEL) (Roche, Mannheim, Germany) staining was performed to show cell death. For colocalization studies, aquaporin 1 (Millipore, Burlington, USA) and aquaporin 2 (Abcam, Cambridge, United Kindom) stainings were co‐stained with TUNEL to distinguish between proximal and distal tubular cell death. IL‐22 stainings were performed as described at different time points after UUO. The extent of tubular injury and interstitial fibrosis was assessed by digital morphometry in ImageJ. To this end, a grid containing 120 (12 × 10) sampling points was used. Grid points overlying the tubular lumen (tubular dilation), atrophic or necrotic tubular cells (tubular cell injury) and interstitial matrix were counted and expressed as a percentage of all sampling points. For CD31 staining, Lectin staining and TUNEL staining, threshold from ImageJ was used to quantify the percentage of positive area per side. For IL‐22 staining, positive cells in the fields were counted. 9 fields from each kidney were randomly selected. All assessments were performed by an observer blinded to the experimental condition.

### Mouse total RNA isolation, cDNA preparation, and real‐time quantitative RT‐PCR

Mouse total RNA was isolated from kidneys stored in RNA later solution after sacrifice and RNA was isolated from an equal amount of tissue mass using a RNA extracting kit (life Technologies, Germany) as described (Sayyed et al. 2010; Weidenbusch et al. 2017). RNA concentrations were measured with NanoDrop 1000 Spectrophotometer. After quantification, RNA quality was assessed via MOPS gels. From isolated RNA, cDNA was prepared by Superscript II reverse transcription (Thermo Fisher) following the manufacturer's instructions as described (Lech et al. 2012). Real‐time quantitative RT‐PCR was performed using SYBRGreen PCR master mix and analyzed with a Light Cycler 480 (Roche Diagnostics) as described. All gene expression values were normalized by 18s rRNA as a housekeeping gene. Double distilled H_2_O was used as negative control for target and housekeeper genes. All primers were purchased from Metabion (Metabion, Planegg, Germany) and sequences are listed in Table 1.

**Table 1:** Murine primer sequences

Murine	Forward (5′‐3′)	Reverse (5′‐3′)
18s	GCAATTATTCCCCATGAACG	AGGGCCTCACTAAACCATCC
CASP1	TCAGCTCCATCAGCTGAAAC	TGGAAATGTGCCATCTTCTTT
CASP8	ATGGCTACGGTGAAGAACTGC	TAGTTCACGCCAGTCAGG
COL1A1	ATGTTCAGCTTTGTGGACCTC	TCATAGCCATAGGACATCTGG
FADD	CACACAATGTCAAATGCCACCTG	TGCGCCGACACGATCTACTGC
KIM1	TGGTTGCCTTCCGTGTCTCT	TCAGCTCGGGAATGCACAA
IL22	TGGGATTTGTGTGCAAAAGCA	TAATTTCCAGTCCTGTCTTCTG
NGAL	ATGTCACCTCCATCCTGG	GCCACTTGCACATTGTAG
SSeCKs	TGAAGCAATCCACAGAGAAGC	CTCATCAAACACTTCCGTTGC
TIMP2	GCAACAGGCGTTTTGCAATG	AGGTCCTTTGAACATCTTTATCTGC
Transgelin	AGCGGACACTAATGAACCTGGG	ACTGGTTGTCCGAGAAGTTCCG

### Protein isolation and western blotting

Total protein was extracted from tissue lysates and processed for Western blotting using RIPA buffer (Sigma‐Aldrich, Taufkirchen, Germany) with protease inhibitors (Roche Diagnostics, Penzberg, Germany) and phosphatase inhibitor (Sigma‐Aldrich) as described (Mulay et al. 2012). Briefly, proteins were separated by SDS‐PAGE and transferred to a polyvinylidene difluoride membrane. To avoid nonspecific binding, membranes were blocked for 2 h at room temperature with 5% BSA in Tris‐buffered saline buffer. Membranes were then incubated overnight at 4°C with primary rabbit antibodies against mouse Bad, p‐Akt, Stat3, p‐stat3 and *β*‐actin (Cell Signaling Technology, Danvers, USA). After washing, membranes were incubated with peroxidase‐conjugated anti‐rabbit IgG secondary antibodies (Cell Signaling) in Tris‐buffered saline buffer and staining was visualized by an enhanced chemiluminescence system (GE Healthcare, Pittsburgh, USA). Staining intensity was quantified using ImageJ.

### Cell culture

The HK2 cell line and K4 cell line were cultured under sterile conditions at 37°C and 5% CO_2_ in medium consisting of DMEM (Gibco/Life Technologies, Grand Island, NY, USA), 10% fetal bovine serum (FBS) (Biochrom, Berlin, Germany) and 1% penicillin/streptomycin (PAA Laboratories, Pasching, Austria).

### Metabolic activity assay

The MTT assay 3‐(4,5‐dimethylthiazol‐2‐yl)‐2,5‐diphenyltetrazolium bromide was performed on HK2 cells and K4 cells to evaluate the metabolic activity induced by IL‐22. Cells were seeded at 5000 cells/well in a 96‐well microculture plate. After cell adhesion for 4 h, various concentrations of recombinant human IL‐22 (rhIL‐22) (Immunotools, Friesoythe, Germany) 1 ng/mL, 10 ng/mL and 50 ng/mL in serum‐free media were added to the wells followed by continuous incubation for 48 h. Cells treated with serum‐free medium only or 10% FBS supplemented media served as negative and positive controls, respectively. Then, 15 *μ*L MTT (5 mg/mL) (Promega, Wisconsin, USA) was added to each well and the plate was incubated at 37°C for another 3 h, after which 100 *μ*L 10% HCl‐SDS was added to each well. The plate was kept at room temperature overnight. Optical density (OD) was quantified via a 96‐well plate reader to record the absorbance at 570 nm. OD is shown after normalization to negative controls.

### Scratch‐induced wound healing assay

HK2 cells and K4 cells were seeded and cultured in 6‐well plates until the wells were confluent with monolayer cells. Then artificial wounds were created with a pipette tip and rinsed twice with PBS to remove the floating cells as described (Kulkarni et al. 2014). rhIL‐22 was added to the cells at different concentrations (1 and 10 ng/mL). Cells treated with medium served as negative controls and treated with 10% FBS served as positive controls. Two pictures per wound were taken on a phase contrast microscope at different time points (0, 12, 24 h). Variations in wound width were analyzed using Image J software.

### Epithelial barrier testing via electric cell–substrate impedance sensing (ECIS)

1 × 10^5^ HK2 cells/well were seeded into ECIS 8‐well arrays (8W1E) (Ibidi, Martinsried, Germany) in serum‐free DMEM overnight in the incubator at 37°C. For primary cell experiments, 4x10^4^ murine isolated primary tubular cells were seeded in ECIS arrays with medium including 10% FBS. Capacitance provides an overall measure of electrode coverage, therefore the value of capacitance is stable at confluence. Wound healing assays were started after confirming confluence by microscopy as well as stable capacitance at 64,000 Hz. We analyzed two types of wound healing assays in HK2 cells. Electric damage was caused by the application of 3 mA at 60 kHz for 60 sec, and the electric fence for growth inhibition was executed every 5 min. Isolated murine primary tubular cells were stimulated with PBS or 20 *μ*g/mL histones at confluency. Six hours later, histones were washed off by exchanging medium, then 1 ng/mL of rmIL‐22 or PBS was added to each well. The value of capacitance just before adding histone and immediately after medium exchange was set as 0 or 1, respectively.

### Statistics

Data are expressed as mean ± SEM, unless specified otherwise in the figure legend. For statistical analysis, one‐way ANOVAs were performed for between group comparisons with post hoc Bonferroni adjustments for multiple comparisons were appropriate. All statistic tests were performed by Prism 6. Significance was assumed at *P* < 0.05.

## Results

### IL‐22 expression during CKD development after UUO surgery

**Figure 1:**
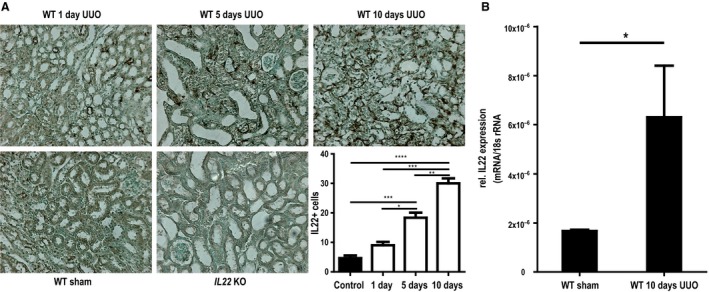
Time course of IL‐22 expression after unilateral ureteral obstruction (UUO). (A) Immunohistochemical IL‐22 staining shows a progressive enrichment of interstitial IL22+ cells after UUO in Balb/C mice (*IL22* ^−/−^ UUO mice from day 5 are shown as negative staining control). (B) IL‐22 gene expression is also significantly upregulated after 10d of UUO in C57/Bl6 mice. WT wild‐type, KO knock‐out *P < 0.05, **P < 0.01, ***P < 0.001.

To explore the changes of IL‐22 expression in the course of chronic kidney disease, we performed renal IL‐22 immunohistochemical staining 1 day, 5 days and 10 days upon UUO in BALB/c mice. While kidney sections from IL‐22 KO mice showed no IL‐22 signal (negative control), a few IL‐22 positive cells were observed in the interstitial compartment of kidneys of healthy BALB/c mice. Interestingly, IL‐22 positive cells accumulated in the interstitium of obstructed kidneys in a time‐dependent manner (Fig. 1A). To assess whether the changes of intrarenal IL‐22 expression were strain‐specific, we performed RT‐qPCR for IL‐22 mRNA expression in C57Bl/6 mice 10 days after UUO. Again, a significant upregulation of IL‐22 was found (Fig. 1B). We concluded that IL‐22 expression increases along with kidney injury and atrophy in the UUO model.

### *Il22* deficiency increases tubular injury upon UUO, but does not affect tubular dilation and interstitial fibrosis

**Figure 2:**
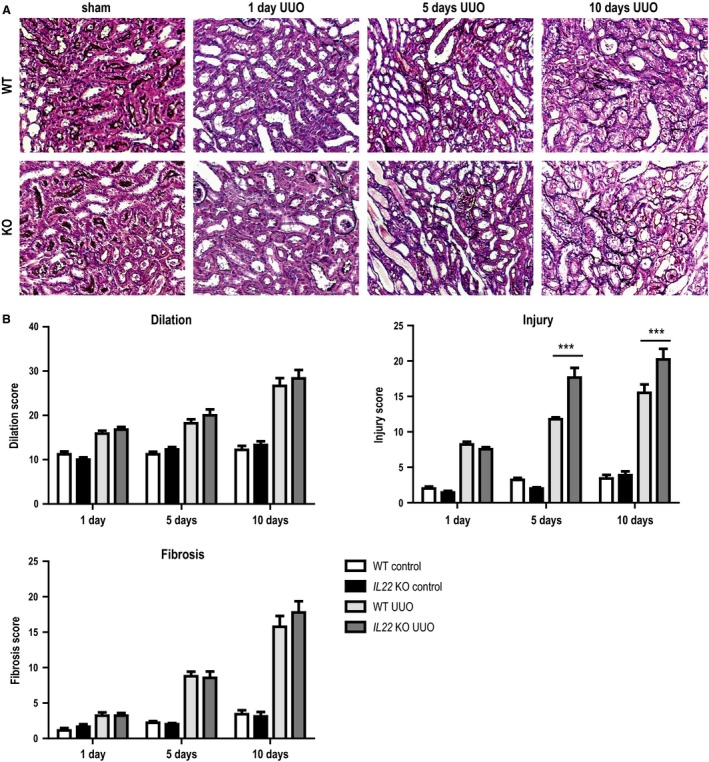
Histopathological changes after UUO in *IL22* ^+/+^ and *IL22* ^−/−^ mice. (A) Representative sections and (B) morphometric scores on tubular dilatation, tubular injury and interstitial fibrosis in *IL22* ^+/+^ and *IL22* ^−/−^ mice after UUO. *** *P* < 0.001.

**Figure 3:**
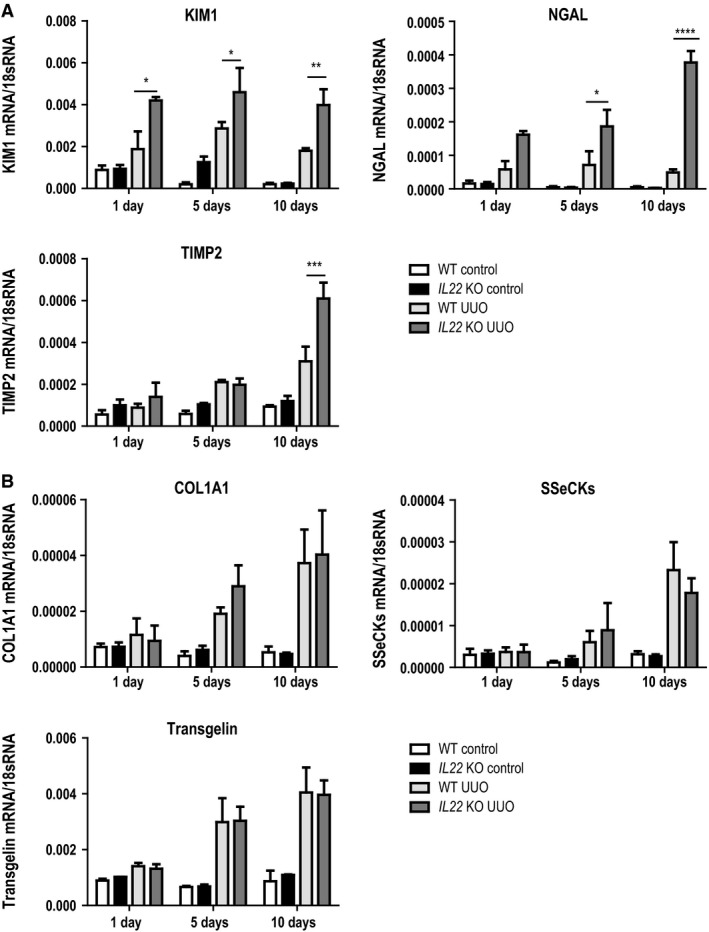
Gene expression of injury and fibrosis markers after UUO. Markers of (A) kidney injury and (B) kidney fibrosis determined by reverse‐transcriptase quantitative PCR (RTqPCR). TIMP2 tissue inhibitor of metalloproteinases 2, NGAL neutrophil gelatinase‐associated lipocalin, KIM1 kidney injury molecule 1, SSeCKs src‐suppressed C‐kinase substrate, COL1A1 collagen type 1 alpha 1. *P < 0.05, **P < 0.01, ***P < 0.001, ****P < 0.0001.

After left‐sided UUO, all mice macroscopically developed hydronephrosis with progressive renal pelvis dilation and thinning of renal parenchyma (not shown). Upon histopathological evaluation by silver staining, we found tubular injury (as indicated by tubular flattening or karyorrhexis) to be significantly increased in *Il22*‐deficient mice at both 5 days and 10 days after UUO surgery compared to wild‐type mice (Fig. 2A and B). Of note, no differences in tubular dilation or interstitial fibrosis were detected between knock out and wild‐type mice (Fig. 2A and B). We concluded that IL‐22 specifically protects tubular epithelial cells from UUO‐induced chronic injury. To further corroborate this hypothesis, we next sought to quantify gene expression changes in UUO kidneys of both *Il22* ^−/−^ and *Il22* ^+/+^ mice by means of RTqPCR. Consistent with the histopathological findings, markers of tubular injury, such as kidney‐injury molecule‐1 (Kim1), neutrophil gelatinase‐associated lipocalin (NGAL), insulin‐like growth factor‐binding protein 7 (IGFBP7) and tissue inhibitor of metalloproteinase 2 (TIMP2) were increased in *Il22* ^−/−^ compared to *Il22* ^+/+^ mice both on day 5 and day 10 after UUO (Fig. 3A), while we could not detect any significant differences in the expression of fibrotic markers such as COL1A1, transgelin or SSeCKs between *Il22* ^−/−^ and *Il22* ^+/+^ mice (Fig. 3B). Taken together, these data show that *Il22* deficiency increases tubular injury upon UUO, but does not affect tubular dilation and interstitial fibrosis.

### *Il22* deficiency leads to loss of proximal tubule cell mass through increased cell death upon UUO

**Figure 4:**
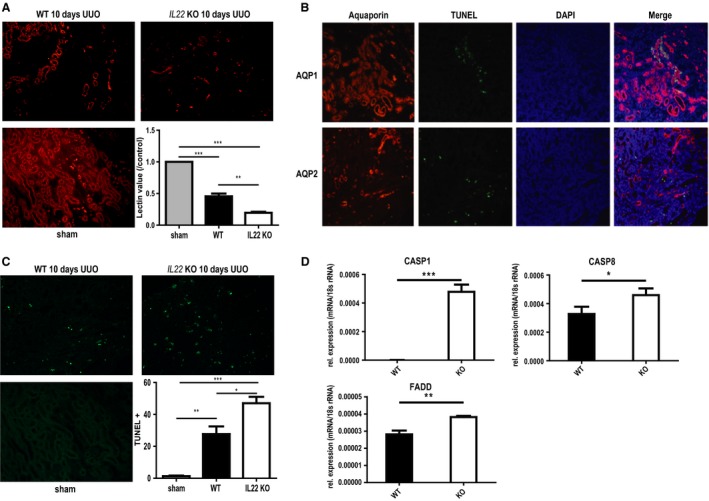
Tubular atrophy and tubular cell death after UUO. (A) Immunofluorescence staining and quantitation of intact proximal tubular cell mass with *Lotus tetragonolobus lectin* in *IL22* ^+/+^ and *IL22* ^−/−^ mice after 10d UUO. (B) *TUNEL (*TdT‐mediated dUTP‐biotin nick end labeling) staining and quantitation of cell death in *IL22* ^+/+^ and *IL22* ^−/−^ mice after 10d UUO. (C) TUNEL (shown in green) co‐immunostaining with aquaporin 1 (shown in orange, upper panel) and aquaporin 2 (shown in orange, lower panel) for localization of dying cells after UUO. (D) RTqPCR‐based gene expression of apoptotic markers in *IL22* ^+/+^ and *IL22* ^−/−^ mice after 10d UUO. CASP caspase, DAPI 4′,6‐Diamidin‐2‐phenylindol, FADD Fas‐associated protein with death domain. *P < 0.05, **P < 0.01, ***P < 0.001.

To further classify the tubular cell phenotype of *IL22*‐deficient animals, we performed *Lotus tetragonolobus* lectin staining to quantify proximal tubule cell mass. As shown in Figure 4A, Lectin positive staining was markedly decreased in *Il22* ^−/−^ mice compared to *Il22* ^+/+^ mice 10 days post‐UUO (80% vs. 54%, respectively; *P* < 0.01). Consistent with increased tubular cell death, there was an increase in TUNEL+ cells observed in *Il22* ^−/−^ mice compared to *Il22* ^+/+^ mice (Fig. 4B). Next, we performed TUNEL co‐stainings with AQP1 and AQP2 to localize proximal and distal tubules in *Il22* ^+/+^ mice, respectively. Interestingly, TUNEL positivity colocalized with AQP1+ proximal tubules exclusively, indicating that indeed increased cell death after UUO was the cause of the marked loss of proximal tubule cell mass in *Il22* ^−/−^ animals (Fig. 4C). As TUNEL positivity is not truly specific for apoptosis (as previously thought), we performed additional gene expression analysis for FADD, CASP8 and CASP1 (Fig. 4D). All markers showed marked increases in *Il22* ^−/−^ mice compared to *Il22* ^+/+^ mice, corroborating the finding of increased tubular cell demise and subsequent tubular atrophy upon UUO in the absence of IL‐22.

### *Il22* activates STAT3 and AKT signaling pathways upon UUO

**Figure 5:**
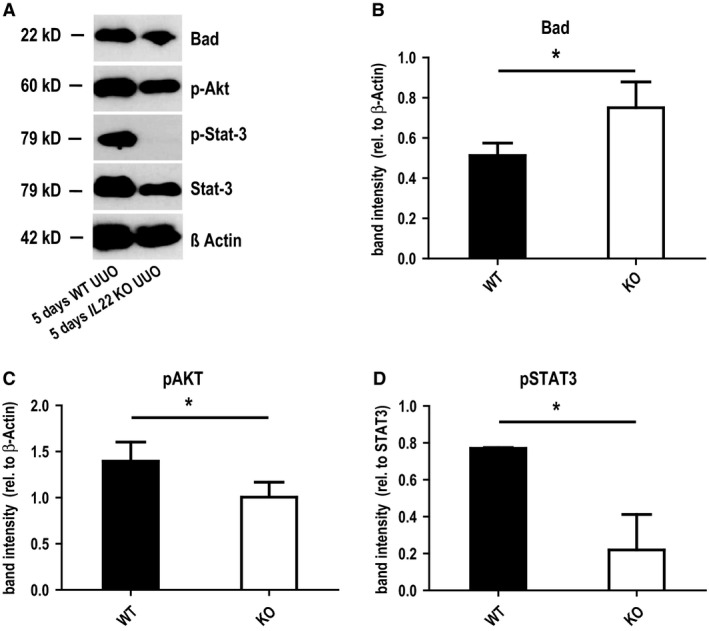
Tissue western blots after UUO in *IL22* ^+/+^ and *IL22* ^−/−^ mice. (A) Gel staining and (B)–(D) staining quantitation of western blots for the apoptotic inducer Bad (B) and IL‐22 receptor downstream signaling mediators STAT3 and Akt (C and D) in *IL22* ^+/+^ and *IL22* ^−/−^ mice after 5d UUO. *P < 0.05.

IL‐22 signaling has been shown to involve the downstream activation of both STAT3 and AKT pathways. Indeed we found decreased phosphorylation of both STAT3 and AKT in UUO kidneys of *Il22* ^−/−^ mice vs. *Il22* ^+/+^ mice at day 5 (Fig. 5A). Consistent with the above‐mentioned finding of increased cell death in *Il22* ^−/−^ mice, we also found increased protein levels of BAD, a proapoptotic mediator and known target of pAKT (Fig. 5B). Taken together, these findings indicate that IL‐22 signaling activates STAT3 and AKT signaling pathways upon UUO.

### *Il22* deficiency does not affect the rarefaction of peritubular microvasculature upon UUO

**Figure 6:**
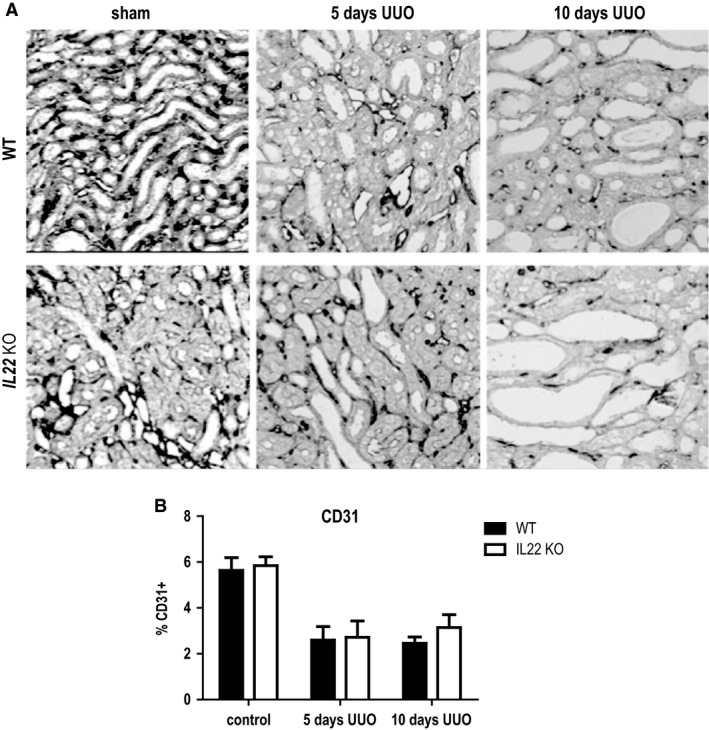
Capillary rarefaction after UUO in *IL22* ^+/+^ and *IL22* ^−/−^ mice. (A) Immunohistochemical CD31 staining and (B) CD31 staining quantitation in *IL22* ^+/+^ and *IL22* ^−/−^ mice after 10d UUO.

To investigate whether IL‐22 plays an additional role on renal endothelium, CD31 staining was performed to analyze vascular rarefaction, which typically accompanies interstitial fibrosis in UUO. Compared with contralateral control kidneys, obstruction of the ureter induced a significant reduction in CD31 expression both at 5 days and 10 days postsurgery (Fig. 6), as expected. Nevertheless, there was no difference of CD31 expression in kidneys dependent on *Il22* genotype, indicating that IL‐22 has no effect on renal endothelial cells.

### IL‐22 enhances proliferation of human tubular cells, but not fibroblasts *in vitro*

**Figure 7:**
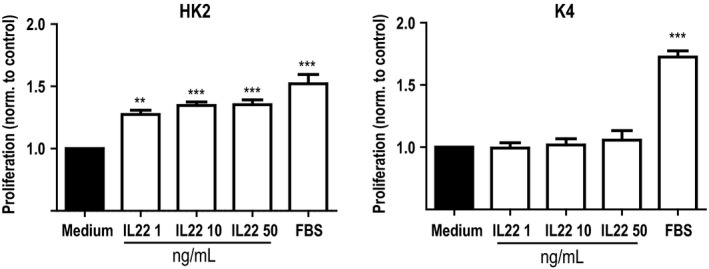
Metabolic effects of IL‐22 on human tubular epithelial cells and fibroblasts. Tetrazolium Reduction (MTT) assays with human tubular epithelial cells (HK2) and human dermal fibroblasts (K4) and increasing doses of recombinant human IL‐22. Significance is indicated for comparison with control. **P < 0.01, ***P < 0.001.

To evaluate if the effects of IL‐22 seen after UUO in mice were transferable to human CKD, we performed experiments with human cells *in vitro*. First, we performed MTT assays in HK2 cells and K4 cells (human proximal tubular cell line and human fibroblast cell line, respectively) to evaluate the effect of IL‐22 on human cell proliferation. After culturing for 24 h, HK2 cells treated with each concentration of rhIL‐22 proliferated remarkably compared with the medium group. Nonetheless, this phenomenon was not observed in K4IM cells, revealing that IL‐22 increased proliferation in an epithelial cell type‐specific manner (Fig. 7).

### IL‐22 enhances migration, re‐epithelialization and barrier function of both murine and human tubular epithelial cells

**Figure 8:**
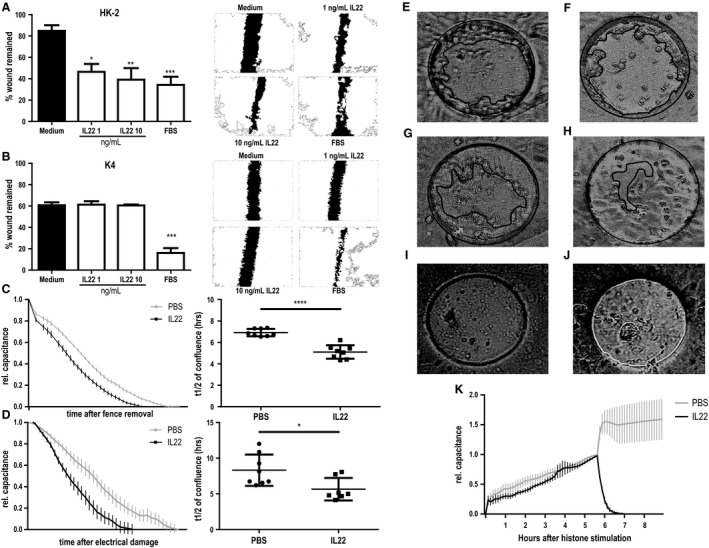
Effects of IL‐22 in a human cell culture model of wound healing. Scratch assays of (A) human tubular epithelial cells (HK2) and (B) human dermal fibroblasts (K4) increasing doses of recombinant human (rh)IL‐22. Left side: Bar graphs for quantitation; right side: representative images for each condition. Significance is indicated for comparison with control. (C–K) Electric Cell‐substrate Impedance Sensing (ECIS) experiments. Capacitance curves (left panel) and capacitance t_1/2_ comparison for vehicle (PBS) and rhIL‐22 treatments of HK2 cells in C) fence removal and D) electrical damage experiments. (E–H) Photographs of ECIS device just after removing fence (E and F) or 5 h after removing fence (G and H). HK2 cells were treated with PBS (E and G) or rhIL‐22 (F and H). Five hours later, wound is smaller in rhIL‐22 treated well (H) than PBS treated well (G). (I and J) Photographs of ECIS device 4 h after exchanging medium with cells treated with vehicle (I) or rmIL‐22 (J). (K) Capacitance curves for histone stimulation and subsequent vehicle (PBS) and rhIL‐22 treatment of primary murine tubular epithelial cells. Note that no t_1/2_ can be calculated for vehicle treatment. *P < 0.05, **P < 0.01, ***P < 0.001.

To mimic epithelial monolayer injury and re‐epithelialization, we performed mechanical scratch assays of HK2 cells and K4 cells in the presence or absence of IL‐22. In HK2 cells, IL‐22 enhanced wound closure after 24 h in a dose‐dependent manner (Fig. 8A), while no such effect was seen in scratch assays with K4 cells (Fig. 8B). To further characterize the effect of IL‐22 on tubular epithelial cell barrier function, ECIS assays were performed allowing online monitoring of renal epithelial monolayers. As described in methods, t_1/2_ was used to measure the effect of IL‐22 on migration and proliferation capacities. Consistent with the results of scratch assays, t_1/2_ in fence experiments, which mimic wound closure, was significantly shorter after rhIL‐22 treatment (Fig. 8C, E–H), confirming an IL‐22‐induced increase in cell migration and proliferation. To analyze the role of IL‐22 on recovery after injury, electrical damage was executed to confluent HK2 cell monolayers. Again, IL‐22 treatment shortened t_1/2_ compared to vehicle (Fig. 8D), indicating that IL‐22 facilitates recovery after injury in human tubular cells. To further examine the role of IL‐22 on recovery after kidney injury, murine primary tubular cells were stimulated with histones, which is released from dying tubular cells after kidney injury, then directly damages tubular cells, and promotes inflammation (Allam et al. 2012). The results showed that cells treated with IL‐22 became confluent within 4 h after removal of histones, while cells treated only with PBS did not show any regrowth during that time (Fig. 8I–K), suggesting that IL‐22 enables recovery after kidney injury.

## Discussion

We had hypothesized that intrarenal leukocyte‐derived IL‐22 would augment tubule integrity in progressive obstructive nephropathy. Indeed, this study shows that IL‐22+ cells increasingly accumulate in the renal interstitium upon UUO. Furthermore, absence of IL‐22 involves more tubular injury, tubular cell death and tubular atrophy, while renal fibrosis remains unaffected *in vivo*. Finally, IL‐22 specifically promotes the metabolic activity, reepithelialization, and barrier function of human tubular epithelial cells, but not fibroblasts in vitro.

It is well known that, upon both acute and chronic renal injury, leukocyte recruitment to the injured kidney regulates both inflammation and regeneration (Anders et al. 2003; Vielhauer et al. 2010; Jang and Rabb 2015). While multiple leukocyte mediators have been shown to be involved in inflammatory processes (Vielhauer and Anders 2009), recently we and others have identified proregeneratory factors secreted by leukocytes after acute renal injury (Kulkarni et al. 2014; Xu et al. 2014). This study now shows the role of one such proregeneratory factor, namely IL‐22, beyond acute kidney injury in chronic progressive obstructive nephropathy.

IL‐22, which is absent from healthy kidneys, is increasingly expressed in the tubulointerstitium of chronically injured kidneys. Consistent with its known role in epithelial cells of other organs (Radaeva et al. 2004; Hanash et al. 2012; Pociask et al. 2013) and after acute kidney injury Kulkarni et al. 2014; Xu et al. 2014), IL‐22 acts as an epithelial cell survival factor in chronic obstructive nephropathy. Of note, the prosurvival effects of IL‐22 are highly specific on proximal tubular cells, while fibroblasts are not affected by IL‐22. This is in line with the previous finding of IL22 receptor expression being confined to epithelial cells, but not immune cells or fibroblasts (Sonnenberg et al. 2011). These cell‐type specific effects of IL‐22 in the kidney disconnect the usually observed tight connection of tubular atrophy and interstitial fibrosis (Bohle et al. 1979; Mackensen‐Haen et al. 1981; Bohle et al. 1987).

Our data which link the prosurvival effects of IL‐22 on renal tubular epithelial cells during chronic injury to the activation of STAT‐ and AKT‐dependent pathways are in line with findings from other organs (Brand et al. 2006, 2007; Mitra et al. 2012). Also in chronic kidney disease, involvement of these pathways has been shown (Tang et al. 2013b; Wiezel et al. 2014). Especially for the case of EGFR‐dependent AKT activation there has been a lot of controversy: while several studies found amelioration of kidney injury through AKT activation (Sinha et al. 2004; Chen et al. 2012; Jang et al. 2012; Kalmar‐Nagy et al. 2013; Ghosh et al. 2016; Mohamed et al. 2017), other studies showed deleterious effects (Bollee et al. 2011; Tang et al. 2013a; Yamamoto et al. 2017). For example, Yamamoto et al. (2017) found recently that the inhibition of AKT signaling by the erlotinib lead to amelioration of the phenotype in a rat model of chronic kidney disease. This effect was found to be driven at least partly via suppression of mesangial cell and macrophage activation (Yamamoto et al. 2017). The findings of this study, however, show that IL‐22‐dependent AKT activation is beneficial in chronic kidney disease via increasing tubular cell survival while other cell types are not affected. Because of its cellular specificity, IL‐22 driven AKT activation which is confined to tubular cells offers new options for highly specific therapeutical interventions compared to, for example, erlotinib.

Unfortunately, the UUO model does not allow for the assessment of systemic effects of uremia and the role of IL‐22 in this context. While usually in CKD both kidneys are diffusely affected by the underlying pathology, the contralateral kidney can fully compensate renal function upon UUO, hence preventing the occurrence of uremia. Also in this study we did not assess the exact cellular source of IL‐22. While we have previously shown, that myeloid cells are the source of intrarenal IL‐22 productions, lymphoid cells (such as T cells and ILCs) have been shown to be the main IL‐22 producers in other organs.

Taken together, we show here for the first time a protective role of IL‐22 in chronic obstructive nephropathy. Specifically, IL‐22 augments tubular cell integrity and epithelial barrier function, but does not affect vascular rarefaction or renal fibrogenesis. It is tempting to speculate that the effects seen in obstructive nephropathy can be extrapolated to other forms of chronic kidney diseases, making IL‐22 a potential therapeutical option to specifically target tubular epithelial cells.

## Conflicts of Interest

None of the authors has a conflict of interest to declare.

## Data Accessibility
